# Detection and predictors of anti-SARS-CoV-2 antibody levels in COVID-19 patients at 8 months after symptom onset

**DOI:** 10.2217/fvl-2021-0141

**Published:** 2021-11-12

**Authors:** Jing Guo, Li Li, Qian Wu, Hongwei Li, Yajie Li, Xinwei Hou, Fangfei Yang, Zhonghua Qin

**Affiliations:** ^1^Department of Respiratory Medicine, Haihe Hospital, Tianjin University, Tianjin, 300350, China; ^2^Department of Laboratory, Haihe Hospital, Tianjin University, Tianjin, 300350, China; ^3^Tianjin Institute of Respiratory Diseases, Tianjin 300350, China

**Keywords:** antibody, COVID-19, IgG, IgM, SARS-CoV-2

## Abstract

**Aim:** To determine SARS-CoV-2 specific IgM and IgG levels of patients with COVID-19 at 8 months after symptom onset and to explore the predictors of antibody levels. **Materials & methods:** The magnetic chemiluminescence method was used to measure the antibody levels. Clinical data were collected and analyzed retrospectively. **Results:** A total of 54 patients were enrolled in this study, of whom 59.3% were IgM positive and 96.4% were IgG positive. The multiple linear regression analysis revealed that the duration of RNA shedding, C-reactive protein level and disease severity were independent predictors of IgG levels. **Conclusion:** COVID-19 patients retained long-term viral-specific protective immunity. Disease severity, C-reactive protein level and duration of RNA shedding were related to antibody levels 8 months after symptom onset.

## Lay abstract

This study aimed to detect the levels of antibodies made by the body in response to COVID-19, 8 months after infection. We reviewed the characteristics of 54 patients with a history COVID-19 to find factors that may influence antibody levels. The results showed that 8 months after infection, almost all the patients had sufficient antibody levels to protect them from another episode of COVID-19 and that antibody levels were especially well maintained in those with a history of severe COVID-19.

COVID-19 is an acute respiratory illness caused by a SARS-CoV-2, which was first reported in late 2019 in Wuhan, China. COVID-19 is currently a pandemic and poses a significant threat to human health. The host immune system plays a crucial role in the defense against SARS-CoV-2 infection. During infection, the SARS-CoV-2 N protein and S protein stimulate an immune response that leads to the production of antibodies [1]. SARS-CoV-2 specific IgM appears within 1 week after infection. The IgM titer rises gradually in the early stage of the disease [2,3] and peaks approximately 3 weeks after symptom onset and then begins to decrease [3–5]. While, IgG levels continue to increase for more than 3 weeks after symptoms onset [4,5]. Recently, some studies have reported stable antibody immunity 6 months after the SARS-CoV-2 infection [6,7]. However, data on the evolution of antibody levels beyond the 6 months is limited and only a few recent studies have documented the durability of the immune response. Reports suggest that the level of IgG remains detectable 8–12 months after the SARS-CoV-2 infection, but that there is a significant drop in the antibody titers compared with the peak levels [8–10].

The magnitude of the antibody response to SARS-CoV-2 infection is heterogeneous between individuals, and the source of much of the heterogeneity in immune memory to SARS-CoV-2 is unknown [9]. Few studies have comprehensively assessed the possible factors influencing the durability of the immune response through multifactorial analysis. It is necessary to have data from multiple locations worldwide, with participants of varying ethnicity, to evaluate the persistence of antibody responses. We assessed the serum-specific IgM and IgG antibody levels of COVID-19 patients 8 months after symptom onset. In addition, we conducted an exploratory analysis to identify factors affecting antibody levels using clinical and laboratory data.

## Materials & methods

### Patients

All participants had been admitted to Haihe hospital in Tianjin, China, with COVID-19 between 21 January and 27 February 2020. All recovered patients were required to retest for SARS-CoV-2 RNA at our hospital 2 and 4 weeks after discharge. After that, the patients were followed-up monthly by community doctors. Follow up consisted of assessing their general condition, symptoms and epidemiological investigation. Those who have recently been to high-risk areas or had a contact history with confirmed or suspected cases were tested for SARS-CoV-2 RNA. None of the study participants were re-infected or exposed to SARS-CoV-2 prior to sample collection for antibody testing. In addition, none of the participants had received a COVID-19 vaccine. The participants were followed up in our hospital between 21 September and 20 October 2020. The inclusion criteria were patients who met the Chinese clinical guidance for COVID-19 pneumonia diagnosis and treatment published by the National Health Commission of China [11]. The exclusion criteria were as follows: patients with diseases of the immune system; patients who died before the follow-up visit; patients who declined to participate; and patients aged less than 18 years.

### Antibody measurement

Venous blood samples were collected from all participants on the day of their 8-month follow-up visit and centrifuged at room temperature. Serum samples were tested for SARS-CoV-2 IgM and IgG antibodies using the magnetic chemiluminescence method. The reagent kits were provided by Bioscience Diagnostic Technology Co., Ltd (Tianjin, China), and the tests were performed according to the manufacturer’s instructions. Antibody levels were expressed as the chemiluminescence signal values divided by the cutoff value (absorbance/cutoff [S/CO]). S/CO values >1.0 were regarded as positive, and tests with S/CO values <1.0 were regarded as negative.

According to the kit instructions, 684 individuals with suspected COVID-19 were included in the clinical trial of this product. The results showed a diagnostic sensitivity and specificity of 88.30% (95% CI: 83.96–91.81%) and 99.50% (95% CI: 98.21–99.94%), respectively, for anti-SARS-CoV-2 IgM; 87.23% (95% CI: 82.77–90.90%) and 99.25% (95% CI: 97.83–99.85%), respectively, for IgG; and 94.33% (95% CI: 90.95–96.72%) and 99.50% (95% CI: 98.21–99.94%), respectively, for a combination of IgM and IgG. The coefficient of variation was used to evaluate the interassay precision. The coefficient of variation of the assay was less than 10%. The performance evaluation of the antibody assay has been described in a previous publication [5].

### Detection of SARS-CoV-2 RNA

Nasal and oropharyngeal swabs were collected every other day after clinical remission of symptoms during hospitalization, and tested for SARS-CoV-2 RNA by real-time reverse transcription-PCR (qRT-PCR) using novel coronavirus (2019-nCoV) nucleic acid detection kit according to the manufacturer’s protocol (Shanghai BioGerm Medical Biotechnology Co., Ltd, China). The BioGerm kit detects two unique targets in the SARS-CoV-2 genome, including *ORF1ab* and *N* genes. The results were considered to be positive if the cycle threshold (Ct) values of both target genes were ≤38; A single target gene Ct value ≤38 was considered as a presumptive positive result and retesting was required. If the Ct value was ≤38 again on retesting, the result was considered positive. SARS-CoV-2 RNA assays were conducted in our hospital laboratory and were confirmed by Tianjin Center for Disease Control and Prevention. The end of RNA shedding was defined as the occurrence of two consecutive negative qRT-PCR tests on samples collected at least 24 h apart. The duration of RNA shedding was considered as the period from illness onset to the first negative PCR test result.

### Data collection

Data on demographics, underlying comorbidity, blood type, disease severity, laboratory test results and treatment were obtained from the electronic medical record system. Based on the severity of the disease, we divided participants into nonsevere and severe groups. Participants who met any of the following criteria were defined as severe cases: shortness of breath, respiratory rate ≥30 breaths/min; oxygen saturation ≤93% at rest; arterial oxygen partial pressure (PaO_2_)/fraction of inspiration O_2_ (FiO_2_) ≤300 mmHg; >50% lesion progression within 24–48 h on pulmonary imaging; respiratory failure requiring mechanical ventilation; shock; or other organ failure requiring intensive care unit monitoring and treatment [11]. Cases that did not met the criteria for classification as a severe case were classified as nonsevere cases. The laboratory results included leucocyte count, neutrophil count, lymphocyte count, C-reactive protein (CRP) and IL-6 within 24 h after admission. We analyzed the data retrospectively. All data were reviewed by two researchers independently.

### Statistical analysis

All of the data were analyzed using SPSS, version 26.0 (IBM Corp., NY, USA). The Kolmogorov–Smirnov test was used to test for normal distribution. Normally distributed continuous variables were reported as means and standard deviations and non-normally distributed variables were reported as the median and interquartile range (IQR). Categorical variables were expressed as numbers and percentages. Continuous variables were analyzed using the Mann–Whitney U test or Kruskal–Wallis H test in the univariate analysis. Fisher’s exact test or Chi-Square test were used for comparisons of categorical variables. The correlation of IgG levels with laboratory profiles was analyzed using the Spearman correlation coefficient. Statistically significant factors in univariate analysis and factors correlated with IgG levels were used as independent variables. IgG levels were log-transformed to achieve a normal distribution and used as the dependent variables. Multiple linear regression was applied to determine the main predictive factors of IgG level. A p-value of less than 0.05 was considered statistically significant.

## Results

**Figure 1.**
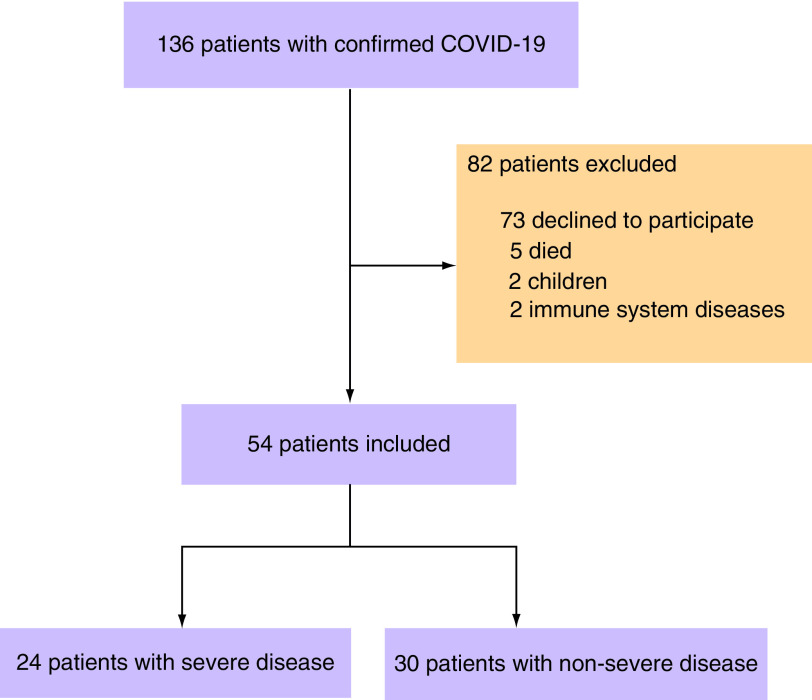
Flow chart of patients with COVID-19 admitted to Tianjin Haihe Hospital between 21 January and 27 February 2020.

A total of 136 patients with COVID-19 were admitted to hospital between 21 January and 27 February 2020. A total of 82 patients were excluded (Figure 1) Five patients died (three during hospitalization and two after discharge); two patients had immune system diseases (one with adult Still’s disease and the other with autoimmune hepatitis); two patients were children; and 73 patients were lost to follow up or declined to participate. About 54 patients were enrolled in this study and had a follow-up antibody test performed between 21 September and 20 October 2020. The mean age of the participants was 48.6 ± 15.5 years and 57.4% were males. There were 24 participants (44.4%) with severe disease and 30 participants (55.6%) with non-severe disease. A total of 19 participants (35.2%) had at least one underlying comorbidity, such as hypertension, diabetes, coronary heart disease or chronic liver disease. In the ABO blood system, blood group A (38.9%) was the most frequent phenotype, followed by B (29.6%), O (16.7%) and AB (14.8%). About 13 participants (24.1%) were treated with glucocorticoids during hospitalization, and one participant with sever disease (1.9%) was treated with intravenous immunoglobulin. The mean interval from onset of the disease to antibody testing was 247.5 ± 11.2 days. The mean length of hospital stay was 16.4 ± 7.6 days, and the mean duration of RNA shedding was 14.1 ± 7.6 days. The detailed participant characteristics are shown in Table 1.

**Table 1.** Baseline characteristics of 54 COVID-19 patients.

	n	Percentage
**Sex**		
Male	31	57.4
Female	23	42.6

**Age (years)**		
<60	43	79.6
≥60	11	20.4

**Disease severity**		
Severe	24	44.4
Non severe	30	55.6

**Comorbidity**		
Hypertension	12	22.2
Diabetes	8	14.8
Coronary heart disease	4	7.4
Chronic liver disease	1	1.9

**Blood type**		
A	21	38.9
B	16	29.6
AB	8	14.8
O	9	16.7

**Glucocorticoid treatment**		
Yes	13	24.1
No	41	75.9

**Immunoglobulin treatment**		
Yes	1	1.9
No	53	98.1
Mean time from symptom onset to antibody test (days) ± SD	247.5 ± 11.2	
Mean length of hospital stay (days) ± SD	16.4 ± 7.6	
Mean duration of RNA shedding (days) ± SD	14.1 ± 7.6	

SD: Standard deviation.

A total of 32 participants (59%) tested positive for SARS-CoV-2 IgM antibody, with a median S/CO value of 1.43 (IQR: 0.43–4.24). A total of 52 participants (96%) tested positive for SARS-CoV-2 IgG, with a median S/CO value of 10.55 (IQR: 6.22–19.80). About 53 participants (98%) were IgM and/or IgG positive, and only one participant tested negative for both IgM and IgG.

### Comparison of clinical characteristics between IgM-positive group & IgM-negative group

As shown in Table 2, the distribution of age, sex, comorbidity, blood type, disease severity and glucocorticoid treatment did not differ between the IgM-positive group and the IgM-negative group (p ≥ 0.05). Additionally, there were no significant differences between them regarding any of the laboratory parameters and the duration of RNA shedding (p ≥ 0.05).

**Table 2.** Comparison of clinical characteristics between IgM-positive group and IgM-negative group.

Parameters	IgM-positive group (n = 32), n (%)	IgM-negative group (n = 22), n (%)	Z*/χ^2^*	p-value
Sex			0.043^†^	0.836
Male	18 (56.3)	13 (59.1)		
Female	14 (43.8)	9 (40.9)		
Age (years)			-^‡^	0.100
<60	28 (87.5)	15 (68.2)		
≥60	4 (12.5)	7 (31.8)		
Disease severity			0.015^†^	0.901
Severe	14 (43.8)	10 (45.5)		
Non severe	18 (56.3)	12 (54.5)		
Comorbidity			1.717^†^	0.190
Yes	9 (28.1)	10 (45.5)		
No	23 (71.9)	12 (54.5)		
Blood type			-^‡^	0.681
A	14 (43.8)	7 (31.8)		
B	8 (25.0)	8 (36.4)		
AB	4 (12.5)	4 (18.2)		
O	6 (18.8)	3 (13.6)		
Glucocorticoid treatment			0.208^†^	0.648
Yes	7 (21.9)	6 (27.3)		
No	25 (78.1)	16 (72.7)		
Duration of RNA shedding (days)	13 (7–18.75)	13.5 (10–21.25)	-0.828^§^	0.407
Leucocyte count × 10^9^/l	4.82 (3.64–6.22)	4.34 (3.25–4.70)	-1.796^§^	0.073
Neutrophil count × 10^9^/l	3.16 (2.44–4.24)	2.66 (1.99–3.42)	-1.391^§^	0.164
Lymphocyte count × 10^9^/l	0.93 (0.69–1.58)	0.90 (0.72–1.23)	-0.784^§^	0.433
CRP (mg/l)	10.75 (2.30–42.09)	9.64 (2.57–30.68)	-0.475^§^	0.635
IL-6 (pg/ml)	6.50 (2.78–21.35)	11.70 (6.78–32.75)	-1.779^§^	0.075

Continuous variables were described as median (Interquartile range). Categorical variables are expressed as numbers (%).

† Chi-Square test;

‡ Fisher’s exact test;

§ Mann–Whitney U test.

CRP: C-reactive protein.

### Analysis of the factors predicting IgG antibody levels

**Figure 2.**
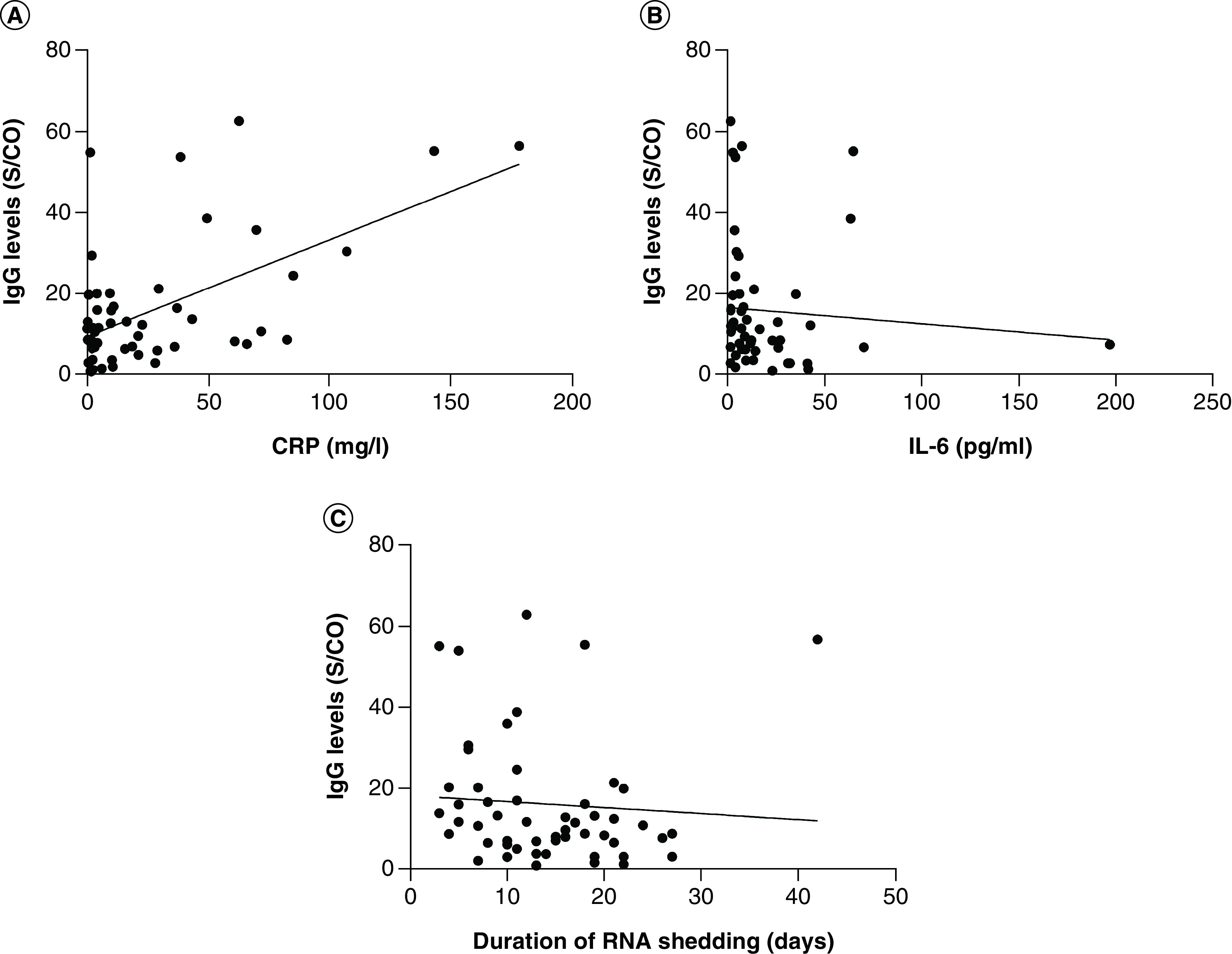
Analysis of the factors related to IgG. **(A)** The correlation between IgG levels and CRP. **(B)** The correlation between IgG levels and IL-6. **(C)** The correlation between IgG levels and the duration of RNA shedding. CRP: C-reactive protein; S/CO: Absorbance/cutoff.

Univariate analysis showed that IgG levels were significantly higher in in participants with severe disease than in those with non-severe disease (median [IQR]: 15.76 [9.50–35.76] vs 6.87 [2.90–13.12]; p = 0.001). In addition, higher IgG levels were detected in participants with normal IL-6 levels than in those with increased IL-6 (median [IQR]: 13.29 [7.56–25.70] vs 7.86 [2.90–12.20]; p = 0.013). There were no significant differences in IgG antibody levels based on sex, age, blood type, comorbidity and glucocorticoid treatment (Table 3). Spearman correlation analysis showed that IgG levels were positively correlated with CRP (*r_s_* = 0.384; p = 0.004) (Figure 2A) and negatively correlated with IL-6 (*r_s_* = -0.301; p = 0.028) (Figure 2B). No correlation was observed between other laboratory parameters (such as leucocyte count, neutrophil count and lymphocyte count) and IgG levels. In addition, there was a negative correlation between IgG level and the duration of RNA shedding (*r_s_* = -0.272; p = 0.049)(Figure 2C).

**Table 3.** Univariate analysis of IgG levels in COVID-19 patients.

Variables	IgG level	Z*/H*	p-value
Sex		-0.487^†^	0.626
Male	10.49 (5.92–19.99)		
Female	11.95 (6.76–19.82)		
Age (years)		-1.023^†^	0.306
<60	10.49 (4.82–19.99)		
≥60	11.87 (8.34–24.47)		
Disease severity		-3.392^†^	0.001
Severe	15.76 (9.50–35.76)		
Non severe	6.87 (2.90–13.12)		
Comorbidity		-1.071^†^	0.284
Yes	11.87 (8.05–20.28)		
No	8.59 (4.82–16.79)		
Blood type		2.870^‡^	0.412
A	13.02 (9.00–20.44)		
B	7.64 (3.93–18.15)		
AB	8.14 (5.92–29.43)		
O	8.60 (2.89–16.13)		
Glucocorticoid treatment		-1.385^†^	0.166
Yes	12.62 (8.37–29.36)		
No	9.53 (5.10–19.00)		
Duration of RNA shedding (days)		-1.197^†^	0.231
<14.1	13.02 (6.12–26.94)		
≥14.1	8.59 (6.52–12.87)		
Leucocyte count (4.0–10.0 × 10^9^/l)		-0.678^†^	0.498
<4	12.41 (7.66–18.78)		
≥4	9.50 (5.37–20.60)		
Neutrophil count (2.0–7.5 × 10^9^/l)		-1.091^†^	0.275
<2	14.36 (7.83–19.80)		
≥2	9.50 (5.92–20.06)		
Lymphocyte count (0.8–4.0 × 10^9^/l)		-1.486^†^	0.137
<0.8	12.62 (7.82–27.88)		
≥0.8	9.04 (3.93–18.74)		
CRP (mg/l)		-1.477^†^	0.140
<10	9.53 (3.45–15.80)		
≥10	12.20 (6.83–30.40)		
IL-6 (pg/ml)		-2.495^†^	0.013
<10	13.29 (7.56–25.70)		
≥10	7.86 (2.90–12.20)		

† Mann–Whitney U test;

‡ Kruskal–Wallis H test.

The normal concentration of CRP is less than 10 mg/l; the normal concentration of IL-6 is less than 10 pg/ml.

CRP: C-reactive protein.

The severity of the disease, CRP, IL-6 and the duration of RNA shedding were included in a multiple linear regression model, which is summarized in Table 4. The data of IgG levels were log transformed. The multiple linear regression analysis revealed that the duration of RNA shedding, CRP and the disease severity were predictive of IgG levels. The results of the regression indicated that the predictors explained 39.8% of the variance (adjusted *R*^2^ = 0.398; *F* = 9.578; p < 0.001).

**Table 4.** Multivariable linear regression analysis of the factors predicting IgG antibody levels.

Independent variable	Coefficient	Standard error	Beta	t	p-value
Disease severity	0.300	0.107	0.341	2.795	0.007
Duration of RNA shedding (days)	-0.017	0.007	-0.300	-2.614	0.012
CRP (mg/l)	0.005	0.001	0.443	3.506	0.001
IL-6 (pg/ml)	-0.002	0.002	-0.170	-1.522	0.135

CRP: C-reactive protein.

## Discussion

After the SARS-CoV-2 virus invades the human body, B cells are stimulated and mature into plasma cells, which produce specific IgM and IgG antibodies to fight off the virus [1]. In general, IgM antibody appears early but is short lived, disappearing within approximately 2 months [12]. Our study showed that the prevalence of specific IgM antibodies was 60% in COVID-19 patients 8 months after symptom onset, which is higher than that of several common viral infections. Maine *et al.* [13] found that the prevalence of IgM antibodies was 30.8% 3 months after infection, but the IgM persisted beyond 3 months in a small proportion of individuals. In a study of SARS, which is also caused by a coronavirus, 36% of the patients were still positive for antinucleocapsid protein IgM antibody 240 days after symptom onset [14] and some patients had a sustained IgM antibody response. However, the high prevalence of IgM positivity observed in our study may be related to the single detection method and the small sample size. The median S/CO value of IgM levels was 1.43. Some of the results were weak positive results because they were close to the cutoff value, and may have been false-positive results. We did not find any significant differences in clinical and laboratory data between the IgM-positive and IgM-negative participants. The durability of IgM antibodies might be associated with interindividual differences. This requires further investigation.

IgG antibodies tended to persist. Previous studies of SARS have shown that the seroprevalence of specific-IgG antibodies was 100% at day 240 after symptom onset, and that IgG antibodies were maintained for an average of 2 years and reduced significantly in the 3rd year [14,15]. Our study showed that the prevalence of specific IgG antibodies was 96.4%, indicating that the participants still had humoral immunity against SARS-CoV-2 8 months after infection. Long *et al.* [16] observed a decrease in specific antibodies 2–3 months after infection in COVID-19 patients, and 40% of individuals with asymptomatic SARS-CoV-2 infection became negative for IgG in the early convalescent phase, suggesting that COVID-19 patients might not have longterm protective immunity. Kaneko *et al.* [17] provided a mechanistic explanation for the limited durability of antibody responses in individuals with SARS-CoV-2 infections, hypothesizing that this was related to the loss of Bcl-6-expressing T follicular helper cells and germinal centers in COVID-19 patients. As time goes by, researchers are learning more about the durability of the antibody response to SARS-CoV-2. A few recent studies have shown that humoral immunity persists for more than 12 months after the onset of symptoms, despite a decline in antibody concentrations [8,10]. Understanding the durability of antibodies to SARS-CoV-2 may improve understanding of the effects of vaccination.

Multivariable linear regression analysis showed that the severity of the disease could independently affect IgG levels. Some studies of the early antibody response at the onset of COVID-19 have shown that IgG levels in patients with severe disease were significantly higher than those with non-severe disease [3,18,19]. It can be seen from this study that the influence of disease severity on IgG antibody levels can last for approximately 8 months. The higher levels of IgG antibodies in patients with severe disease might be attributable to a stronger stimulation of B cells and formation of longer lived plasma cells [20]. The persistence of relatively high levels of IgG antibodies suggests that humoral immunity persists in patients with severe COVID-19. Some studies have shown that males have higher antibody responses than females in the early stage of disease [8,21]. This may be because males with COVID-19 tend to have more severe disease than females, and the enhanced inflammatory responses could drive higher B cell recruitment and more antibody production [21]. However, in our study the difference between males and females did not persist 8 months after infection. This result is consistent with what has been found in a recent study [8]. Sex-related differences in the immune response warrants further study.

Our study also found that there was a correlation between CRP, IL-6 and IgG levels. CRP is a blood marker that indicates the overall degree of inflammation in the body. The CRP levels in the patients with severe disease tends to be higher than those with milder disease [22]. Gozalbo-Rovira *et al.* [23] observed weak correlation between the antibody assays and CRP levels. Our results suggested that this correlation may be persist for several months. In addition, there was a negative correlation between IL-6 and IgG levels. This may be because a large number of cytokines inhibit the production of memory B cells in the early stage of the disease, thus affecting the long-term protective effect of specific antibodies [17]. However, the effect of IL-6 was not significant in the multivariate analysis. It appears that the magnitude of the antibody responses and the state of inflammation in COVID-19 patients are related. This relationship requires further in-depth study.

This study also found a significantly negative correlation between IgG levels and the duration of RNA shedding. Some factors that may delay the SARS-CoV-2 nucleic acid tests turning negative include hypoalbuminemia, underlying coronary heart disease and corticosteroid treatment, but the main determinant of the time that it takes for SARS-CoV-2 nucleic acid tests to revert to negative it is more likely to be related to the autoimmune response [24–26]. Ling *et al.* [25] showed that a longer duration of virus shedding was associated with a lower absolute value of CD4^+^ T lymphocytes before treatment. The weak immune response of the host against the virus, which leads to a decreased ability of the host to clear the virus, prolongs the duration of RNA shedding [24]. It can be inferred from the above that the long-term effect of antibodies was also weak.

It should be noted that there were some limitations to our study. First, it was performed in a single center and had a relatively small sample size. There were few confirmed COVID-19 cases in Tianjin, China and most patients returned to their normal lives after recovery and refused to participate in long term follow up. Second, although we carried out clinical assessments to determine the health status of participants during the 8-month interval between COVID-19 onset and antibody testing and none of the participants developed symptoms of re-infection or provided a history of exposure, the dynamic changes in the antibody levels were not checked. Last, our study was limited to follow up 8 months after infection and longer-term observations are needed.

## Conclusion

SARS-CoV-2-specific IgG antibodies last for at least 8 months after symptom onset. The severity of COVID-19, duration of RNA shedding and the CRP levels were identified as potential predictors of IgG levels. Some patients also had persistent detectable IgM antibodies 8 months after infection, but no determinants of persistent IgM positivity were identified. Overall, our study results suggest that there is a lasting specific immune response after COVID-19 and helps to promote vaccine development. Mild COVID-19 survivors may still need to be vaccinated.

**Summary points**

- In patients with COVID-19, the duration of immune protection after initial SARS-CoV-2 infection is still unknown; therefore, we measured antibody levels 8 months after symptom onset.
- Of those tested, 59.3% were still SARS-CoV-2 IgM-positive 8 months after infection, which is higher than the persistence of IgM antibodies following other common viral infections.
- Almost all participants were SARS-CoV-2 IgG positive 8 months after infection.
- The duration of RNA shedding, C-reactive protein and disease severity were independent predictors of IgG levels.
- These findings suggest that there is long-term immunity after SARS-CoV-2 infection.
